# Environmental Domains and Range-Limiting Mechanisms: Testing the Abundant Centre Hypothesis Using Southern African Sandhoppers

**DOI:** 10.1371/journal.pone.0054598

**Published:** 2013-01-23

**Authors:** Simone Baldanzi, Christopher D. McQuaid, Stefano Cannicci, Francesca Porri

**Affiliations:** 1 Coastal Research Group, Department of Zoology and Entomology, Rhodes University, Grahamstown, South Africa; 2 Dipartimento di Biologia Evoluzionistica, Università degli Studi di Firenze, Firenze, Italy; University of California, Berkeley, United States of America

## Abstract

Predicting shifts of species geographical ranges is a fundamental challenge for conservation ecologists given the great complexity of factors involved in setting range limits. Distributional patterns are frequently modelled to “simplify” species responses to the environment, yet the central mechanisms that drive a particular pattern are rarely understood. We evaluated the distributions of two sandhopper species (Crustacea, Amphipoda, Talitridae), *Talorchestia capensis* and *Africorchestia quadrispinosa* along the Namibian and South African coasts, encompassing three biogeographic regions influenced by two different oceanographic systems, the Benguela and Agulhas currents. We aimed to test whether the Abundant Centre Hypothesis (ACH) can explain the distributions of these species’ abundances, sizes and sex ratios and examined which environmental parameters influence/drive these distributions. Animals were collected during a once-off survey at 29 sites over c.3500 km of coastline. The ACH was tested using a non-parametric constraint space analysis of the goodness of fit of five hypothetical models. Distance Based Linear Modelling (DistLM) was performed to evaluate which environmental traits influenced the distribution data. Abundance, size and sex ratio showed different patterns of distribution. A ramped model fitted the abundance (Ramped North) and size (Ramped South) distribution for *A. quadrispinosa.* The Inverse Quadratic model fitted the size distribution of *T. capensis*. Beach slope, salinity, sand temperature and percentage of detritus found on the shore at the time of collection played important roles in driving the abundance of *A. quadrispinosa*. *T. capensis* was mainly affected by salinity and the morphodynamic state of the beach. Our results provided only some support for the ACH predictions. The DistLM confirmed that the physical state of the beach is an important factor for sandy beach organisms. The effect of salinity and temperature suggest metabolic responses to local conditions and a role in small to mesoscale shifts in the range of these populations.

## Introduction

The complex dynamic and interlocking effects of climate change on organisms and their environments can lead to dramatic changes in the distribution of species and ultimately, loss of biodiversity [1], [2]. Accordingly, predicting shifts in species ranges and the underlining mechanisms behind such changes, has become a central challenge in conservation biogeography [3].

Range expansion/contraction and distributional shifts occur naturally and continuously, but can be accelerated by changes in climate and by human activities [4], [5] such as pollution, environmental degradation, changes in land use and the introduction of invasive species [5], [6]. Modelling approaches to understanding species distributions have focused most intensively on the description of a bioclimatic envelope that characterises the natural distribution of a species [7]. Such simplification is a necessary response to the complexity of the real world, but a more realistic understanding of species distributions must also include a wide range of abiotic and biotic variables [8]. Such an approach assigns a central role to the spatial domains of natural variables, with climatic variables having a dominant effect from regional to global scales, while other variables, such as biotic interactions, have more localised effects [7], [9]. At regional scales, geographic patterns of abundance are fundamental to ecological issues, providing information on species range limits, gene flow among populations, population dynamics and species’ responses to environmental change [10], [11]. It is widely accepted that the abundances of species are greatest at the centres of their distributional ranges and decline towards the margins [10], [12]–[16]. This concept is the “Abundant Centre Hypothesis” (ACH hereafter). This idea has been explored by several authors [10], [12], [17] and extensively used to understand ecological and evolutionary processes [10], [11]. Nevertheless, the concept remains largely theoretical and empirical evidence for the patterns predicted by the ACH is still weak [18] and equivocal [11], [13], [14], [16]. Sagarin and Gaines [10] reviewed a large number of published works that tested the ACH, and found that only 39% of these supported the ACH, probably because abrupt changes in biotic and/or environmental conditions may result in sharp, rather than gradual gradients in abundance [12], [18]. The need to evaluate variation in abundance at large geographical scales has been stressed by several authors with an emphasis on the need for large numbers of sampling sites, in order to detect the realistic edges of species distributions [10], [14], [16], [19].

Additional features such as genetic structure, physiological proxies, life-history traits or biophysical variables have been used to test the ACH, as such factors can reflect both distributions and range boundaries [14], [20]–[22]. White *et al.* [23] identified several types of relationships between size and abundance, assuming that the size-abundance relationship is a fundamental link between the individual and the population level. Rivadeneira *et al.* [14] linked the distribution of abundance with variation in life history traits, such as sex ratio and the proportion of reproductively active females, concluding that sex ratio provided the strongest support for the ACH, with females being more abundant at the centre and males at the edges. Virgós *et al.* [15], while testing the ACH on the European badger (*Meles meles*), concluded that body size is strongly related to food availability and resources, which are supposed to be higher and of better quality at the centre of distribution and indeed they found individuals were larger at the core than the periphery.

Most tests of the ACH have focused on terrestrial species [15], although there have been some studies of marine systems [14], [16], [19], [21], [24], [25]. Intertidal and supratidal organisms are considered ideal models to test “range-wide hypothesis” (including the ACH) due to the linear geometry of their geographical ranges, reducing it at a one-dimensional pattern of distribution, where edges and centre are relatively easy to define [11].

Here, we investigate the biogeography of two species of southern African sandhoppers (Crustacea, Amphipoda, Talitridae) to test the predictions of the ACH, and to understand the influence of environmental variables on their abundances. How species respond to environmental variability is crucial in sandy beach ecology, as fluctuations in abundance at large spatio-temporal scales are fundamental to how these systems function [24], [26]–[30]. Additional advantages of using this system to test the ACH are that it is particularly strongly forced by environmental factors and experiences little human impact. The effects of the environment on the relation between species range and population declines, are critical to effective tests of the ACH, as these two phenomena are generally correlated [31], [32].

The two study species, *Talorchestia capensis* (Dana, 1853) and *Africorchestia quadrispinosa* (K.H. Barnard, 1916) show different distributions along the sandy shores of Namibia and South Africa, providing multiple tests of ACH predictions along a one-dimensional environmental gradient. *A. quadrispinosa* has a wide North-South distribution, encompassing two biogeographic regions [33], forming an ideal model to test the classic ACH [14], [16]. On the other hand, *T. capensis* has a wide, but patchy distribution, from the west to the east coast of South Africa, encompassing three different biogeographic regions (the cool-temperate west, warm-temperate south and sub-tropical east coasts, [33]), offering a highly diversified model to test the ACH.

We hypothesised that: 1) the geographic variation in abundance, size and sex ratio of these two species of southern Africa sandhoppers, should be explained by the predictions of the ACH. Particularly, we expected a good positive test for *A. quadrispinosa*, since its linear North-South distribution fits well with the classical inferences of the ACH [14], [16]; 2) among the environmental conditions experienced by these animals, the morphodynamic state of the beaches and temperature seem to be the most relevant parameters for a distributional range that extends across different latitudes [29], [34] and are likely to have strong influences on abundances.

## Materials and Methods

This study was carried out in strict accordance with the recommendations in the “permit for the purposes of a scientific investigation or practical experiment in term of section 83 of the Marine Living Resources Act, 1988 (Act no 18 of 1998)”. The Permission has been approved by the Chief Director of Fisheries Research and Development; Department of Agriculture, Forestry and Fishery, Republic of South Africa (Permit ref. no: RES2012/05).

### Study Sites

**Figure 1:**
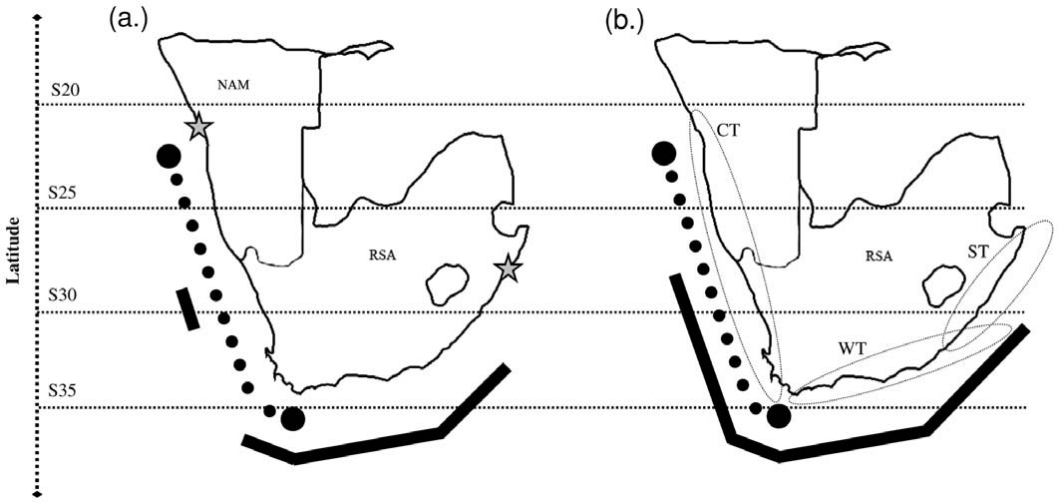
Distributional range of the two species from the field surveys (a) and historical range data of distribution, based on the published data (b). The dotted line represents the range of *Africorchestia. quadrispinosa* and the solid line the range of *Talorchestia. capensis*. The star symbols represent the range of the entire sampling area (a.) The ellipse indicates three biogeographical regions (b): Cool Temperate (CT), Warm Temperate (WT), Sub-Tropical (ST), [33]. The ST region has a transition zone which includes the limit of distribution of *Talorchestia capensis.* Latitudes are reported on the left side of the map.

The study area includes a long coastline encompassing three biogeographic regions: the cool-temperate Namaqua province on the west coast, the warm-temperate Agulhas province on the south coast and the subtropical East Coast province [35], [36]. The sampling area ran from Richards Bay (KwaZulu Natal, East coast, South Africa) to Wlotzkasbaken (West coast, Namibia) (Fig. 1a). The geographical coordinates for each site were taken using a global position system receiver (Etrex, Garmin) and are reported in the Table S1. In order to collect animals at the highest site-resolution possible, we planned to sample sites no more than 100 km apart, based on Google Earth® imagery. Once at a location, we established the best area according to accessibility and beach width as a minimum width was necessary to allow the setting of traps, (see below). Based on this, sandy shores with or without detritus were both investigated. Animals were collected during winter, 2010 (South Africa, from June to August) and 2011 (Namibia, June). Two separate surveys were necessary due to the long distances covered and logistic constraints.

### Study Species

Sandhoppers are semiterrestrial crustaceans in the Order Amphipoda. The Talitridae is the only family including truly terrestrial amphipods and, although many are found close to the sea on the upper parts of the shore, some occur inland [37]. The species investigated in this study were: *Talorchestia capensis* (Dana, 1853) and *Africorchestia quadrispinosa* (K.H. Barnard, 1916).

Sandhoppers burrow into moist sand during the day, avoiding the stresses of heat and desiccation [38], [39] and emerge at night, when the air temperature is cooler, and the risk of predation is reduced [40]–[43]. Numerous studies report a strong link between the diet of sandhoppers and detrital macrophytes (e.g. [44], [45]), although other studies suggest a more complex opportunistic feeding strategy that allows sandhoppers to utilise alternative sources of food, such as diatoms [46] and even conspecifics [47]. Porri and coauthors [48], using stable isotope analysis, found no trophic link between the sandhopper *Talorchestia capensis* and the detritus underneath which animals were found.

### Collection and Laboratory Analysis

Sandhoppers were collected at each site using pit-fall traps that were set up above the high water mark at dusk and emptied the following morning at sunrise, during neap tides. This allowed us to capture sandhoppers that migrated between the intertidal and the supratidal, giving samples that integrated sandhopper abundances across the shore. The sampling unit was made up by four traps (made of half two-litre plastic bottles, filled up with soapy water) set on the four corners of two plastic baffles that were buried crossed into the sand in an ‘X’ arrangement (Figure S1). Two levels were assessed: level 1, (L1) at the Spring Tide High Water Mark (STHWM) and level 2, (L2), at the Neap Tide High Water Mark (NTHWM). Each level included three replicates. Collections from the four corners were pooled to form a single sampling replicate. The three replicates within a level were 50 m apart and the two levels were displaced by 25 m relative to one another: for example, the first replicate of Level 1 (L1a) was displaced by 25 m alongshore relative to L2a. This “chessboard” arrangement allowed us to cover a total area 125 in length and an average of 5 m wide (depending on the tidal range) (Fig. S1). Animals were collected during seaward migration (occurring just after dusk) and landward migration, (at sunrise). After a 12 h collection period, all traps were emptied into 500 µm metal sieves, to collect adults, early juveniles and, when present, eggs. Specimens were stored in 75% ethanol and transported to the laboratory for further analysis. This design was chosen after a preliminary study carried out at four different sites on the south coast of South Africa during which we compared two different methods involving overnight pitfall-traps (which covered an area similar to that described above) and a core-transect method. The latter method included the use of three transects perpendicular to the shore, from the top of the dune to the swash: a core of sand (20 cm depth and 10 cm wide) was taken at each transect, every three meters landward and seaward, starting from the drift line. The sand was sieved immediately using a 1 mm mesh. Since no animals were collected using the core-transect method, we opted for the pitfall traps. Pitfall traps are used to collect sandhoppers worldwide during their nocturnal migration [49], [50].

Animals were identified following Griffiths [51], counted, measured and sexed using a stereomicroscope (32× and 64× magnification). The total body length (size), measured at 8× magnification, was taken from the base of the first antenna to the base of the telson [50]. On the basis of the body length, individuals were grouped into 0.5 mm size classes [50]. Males were distinguished by the presence of an enlarged 2^nd^ gnathopod and genital papillae. Females do not show an enlarged 2^nd^ gnathopod and could be distinguished by the presence of osteogytes. Individuals lacking secondary sex characters were classified as juveniles [50]. Since the identification of juveniles to species was not possible (especially when more than one species was collected at a site), only adults were considered for the analysis of abundance and size (see below).

### Environmental Parameters

Several environmental parameters (temperatures of water and sand, water salinity and percentage cover of detritus on the shore) were recorded during the deployment of traps. Sea temperature at the swash line and sand temperature measured at 10 cm depth for each sampling unit were taken using a mercury thermometer. Sand temperature was recorded twice, at dusk, during the deployment of the traps and at sunrise, during the collection. The double measurements minimised variability due to time of day. Salinity was measured using a handheld refractometer (Atago, S-10E). Percentage of detritus cover was estimated using a grid quadrat (50 cm × 50 cm): ten haphazard measurements were taken along the detritus line. Any organic matter found in the sampling area, under which animals occurred was considered detritus and the percentage was zero if no detritus was found. Several measurements were used to define the physical state of the beach: beach slope, beach width and grain size. We did not measure breaker height at the time of collection, which can be used to define beach morphodynamic type, but which is unreliable and difficult to measure accurately (McLachlan pers. comm., also see [52] for details of the Dean parameter). Instead the description of beach morphodynamic state was based on beach slope, beach width and grain size as measured in the field (McLachlan pers. comm.). Beach slope was measured by two operators using a manual level to detect changes in slope every 3 m from the swash area to the high tide mark. This is a modification of Emery’s method [53]. The beach width was considered as the portion of beach between the swash at low tide and the high tide mark. Sand samples were collected using a core sampler of 3.5 cm in diameter to a depth of about 20 cm. Sand samples were transported to the laboratory for granulometric analysis following a modified Falk and Ward procedure [54]. After analysis, the following indices were calculated: Area, a measure of intertidal area obtained by dividing tide range by the beach face slope [52], Beach Index, similar to Area but including a measure of sand particle size (BI, [52]), Beach Deposit Index (BDI, [55]), an index that does not consider the tidal range. Indices were calculated using the following formulae:

- Area = log×(Tide÷Slope)
- BDI = (1÷tanB)×(a÷Mz)
- BI = (Sand×Tide)÷Slope

where: Tide is the maximum spring tidal range (meters); Slope or tanB is the beach slope; B is the average intertidal Beach slope, a  = 1.03125 is the median grain size of the sand particle size classification; Mz is the average intertidal sand size (mm) and Sand is the mean sand particle size (phi units +1) [52], [56], [57]. The dimensions of the indices are: log meters (Area), log phi·m (BI). BDI is dimensionless.

The morphodynamic state of a beach is well known to have a strong effect on the biota. To ensure that the analyses were not distorted by mixing shores of different states, we categorised each shore based on BI index, following [52]. All shores were classified by this system as ‘Intermediate’ and consequently were included in the analyses.

### Data Analyses

For each site, several sandhopper’ variables were calculated: Absolute Abundance (AbA), Relative Abundance (RA), Relative Size (RS) and Sex Ratio. AbA is the number of individuals reported from the collections obtained by pooling all replicates and levels. RA was obtained by dividing the number of individuals for each site (AbA) by the maximum abundance found at any site within the range [10]. This was done to allow reasonable comparisons among sites and species [14], [17], [19]. RS was calculated by dividing the size of individuals (mm) by the maximum size of any conspecific from any site. A Student t-test was used to assess differences in size between the sexes using data pooled for all sites Sex ratio was calculated as the proportion of males to females (males/females). Chi-squared tests (χ^2^) were used to determine whether sex ratio values differed from the expected 1∶1 ratio.

To test the predictions of the ACH on abundance, size and sex-ratio, a non-parametric constraint space analysis was used, following procedures used by Enquist *et al.* and Sagarin and Gaines [10], [17]. These models are commonly used to describe patterns of abundance of species throughout their ranges [10], [11], [14]. To evaluate whether abundance, size or sex ratio varied with position within the distributional range, a Range Index (RI) was calculated using the expression proposed by Brown and Sagarin and Gaines [10], [58].

- RI = 2×(LS)÷R

where L is the position (i.e. the distance in km) of a location relative to the northern or western range limit, S is the midpoint (in km) of the geographical range, and R is the extent of the geographical range (km). The RI index ranges between −1 and 1, so that sites with values close to 0 are considered to be near the centre of distribution and values close to −1 and 1 are near the western/northern and eastern/southern edges respectively.

The degree of fit of each model (see Figure S2 for a schematic representation) to the observed data was evaluated by calculating the residual sum of squared deviations (RSS) for sites exceeding the constraint boundaries generated by each model. The significance of the observed RSS values was evaluated by generating 1,000,000 randomized values of RI, RA, RS and Sex Ratio. The fit of the model was considered significant when the observed RSS value was lower than the 5th percentile of the randomized distribution. The degree of support for each model was evaluated by calculating the Akaike’s Information Criterion (AIC), selecting all models with Akaike weights >0.25 [10], [11], [14]. Analyses were carried out using a routine in R [59].

All the works referred to above that describe the ACH consider a North-South range of distribution as the position of sites expressed in Latitude [10]. We used a scale of kilometres instead, to adapt the expressions to distributions that follow the South African coast, as was done by Tuya *et al.* [18] for endemic reef fishes of South to Western Australia.

Kilometres were accurately calculated using the Ruler tools in Google Earth® imagery, measuring the distances between sites, from a height of 5 km. The coordinates of the sites recorded in the field (Table S1) were uploaded to assess the exact location of the sampling areas.

A Distance–based Linear Model (DistLM, [60]) procedure was performed to analyse the relationship between the abundance of sandhoppers and environmental traits, physical variables and indices, i.e. sand temperature, water temperature, salinity, percentage of debris coverage, slope, grain size (Mz and Sand), Tide (maximum spring tidal range), Area, BDI and BI. Slope is reported as log(1/Slope) since it is considered for a good predictor for regional patterns of the abundance of sandy beach fauna [52]. Multi-collinearity for the environmental variables was detected between Area and Tide, Area and Slope, Mz and Sand, after examining the Draftsman’s plots [61] and Area and Sand were therefore removed.

For the model used for the DistLM, we selected the AIC (Akaike Information Criterion), basing the analysis on the Bray-Curtis resemblance measure after square root transformation of the abundance data [62]. The data contained a high proportion of zero’s and therefore a dummy variable with a value of 0.0001 was added to the Bray-Curtis similarity matrix to moderate spurious similarities where no species were recorded in two compared samples [61]. All analyses were carried out using PRIMER (ver. 6.1.12) and PERMANOVA+(ver. 1.0.2) [62], [63].

## Results

### Geographical Range and Pattern of Abundance, Size and Sex Ratio

A comparison between historical distribution data and the results from the present manuscript is summarised in Fig. 1a,b.

The most abundant species was *A. quadrispinosa,* with a total of 12496 adults collected. Its highest concentration occurred within the centre of its distribution (from Port Nolloth to Cape Columbine, Table S1). *T. capensis* showed high abundances of individuals (total n  = 8 398 adults), though 90% were collected from a single site (Hondeklipbaai, Table S1). For size and sex ratio, *T. capensis* had the largest animals, with a significant difference between the sexes (males: 10.8±1.4; females: 9.9±1.4; t-test, p<0.0001), but no significance differences in the proportions of females to males. *A. quadrispinosa* individuals were smaller than *T. capensis*, with no difference between males and females (males: 8.8±2.8 mm; females: 8.7±2.7 mm). Significant differences in the proportions of females to males (χ^2^ = 44.36; p<0.005) were observed.

**Figure 2:**
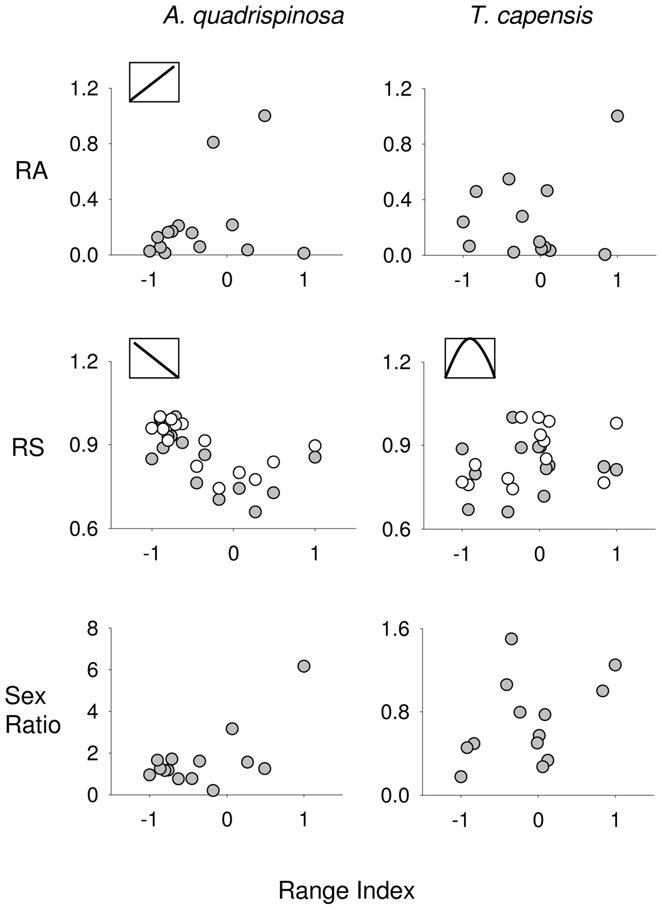
Pattern of geographic distribution of abundance (upper panel), size (middle panel) and sex ratio (lower panel) for the two species of sandhoppers (top of the figure). The range is reported as Range Index (see Material and Methods), where −1 =  southern/eastern range; 0 =  centre; +1 =  northern/western range. The model which best fitted the observed is reported as a small icon for each pattern of distribution. The IQ model (see Material and Methods) is referred to the female size distribution. Ra = relative abundance; RS = relative size; filled dots: males; open dots: females.

The geographic pattern of relative abundance, size and sex ratio differed between the two species with no predominant pattern (Fig. 2). Sex ratio did not fit any of the models for either species. *A. quadrispinosa* showed the best degree of fit with a ramped pattern explaining abundance (Ramped North; Table 1a, left panel; Fig. 2, upper panel) and male and female size (Ramped South; Table 1b, 1c; Fig. 2, middle panel). For *T. capensis*, only the distribution of female size showed a significant fit, with the Inverse Quadratic model (Table 1c, right panel; Fig. 2, middle panel), while abundance, male size and sex ratio did not show any patterns related to any of the tested ACH models.

**Table 1:** Degree of fit of each models for abundance, male size, female size and sex ratio.

	Africorchestia quadrispinosa				Talorchestia capensis			
Model	RSS	5^th^ percentile	AIC	AICwt	RSS	5^th^ percentile	AIC	AICwt
(a) abundance								
No	1.0092	0.2927	−76.24	0.00	0.2412	0.0104	−112	0.00
I.Q.	1.0007	0.0427	−76.46	0.00	0.0715	0.0041	−142.4	0.00
A.E.	0.4974	0.0125	−89.93	0.00	0.1282	0.0495	−123.8	0.00
**R.N.**	0.0701*	0.1385	−142.9	1.00**	0.2402	0.0091	−112.1	0.00
R.S.	1.1308	0.0149	−73.4	0.00	0.0032	0.0010	−220.1	1.00
(b) male size								
No	6.0077	5.2794	−31.65	0.00	2.6125	2.4092	−52.46	0.00
I.Q.	2.6588	2.155	−52.02	0.00	1.9407	1.6826	−59.9	0.19
A.E.	1.6798	1.558	−59.5	0.00	3.4973	2.9577	−41.17	0.00
R.N.	4.8759	3.8633	−36.86	0.00	2.7922	2.5875	−50.8	0.00
**R.S.**	1.2536*	1.5218	−70.82	0.99**	1.7306	1.4005	−62.76	0.80
(c) female size								
No	6.8369	6.1522	−28.41	0.00	2.4942	2.6886	−53.62	0.01
**I.Q.**	3.2122	2.6582	−47.3	0.00	1.7786*	1.868	−62.08	0.88**
A.E.	1.9965	1.9814	−55.19	0.02	4.2328	3.3354	−36.4	0.00
R.N.	5.4417	4.6036	−34.12	0.00	3.0313	2.972	−48.75	0.00
**R.S.**	1.7177*	1.9163	−62.95	0.98**	2.105	1.6629	−57.86	0.11
(d) sex ratio								
No	21.944	17.924	0.7408	0.00	0.3592	0.2792	−102.1	0.00
I.Q.	14.25	12.194	−10.05	0.44	0.0396	0.0541	−157.2	1.00
A.E.	13.101	11.231	−8.155	0.17	1.0152	0.2877	−72.1	0.00
R.N.	17.362	16	−5.114	0.04	0.712	0.3373	−84.96	0.00
R.S.	14.507	10.824	−9.606	0.35	0.1393	0.0412	−125.8	0.00

* Significant values for RSS.

** higher degree of support for each model (AICwt>0.25).

Bold: fitted model.

No = Normal; I.Q. = Inverse Quadratic; A.E. = Abundant Edges; RN = Ramped North; RS = Ramped South. RSS = residual sum of square; AIC = Akaike Information Criterion; AICwt = AIC weight.

### Environmental Domains Driving Abundance

**Figure 3:**
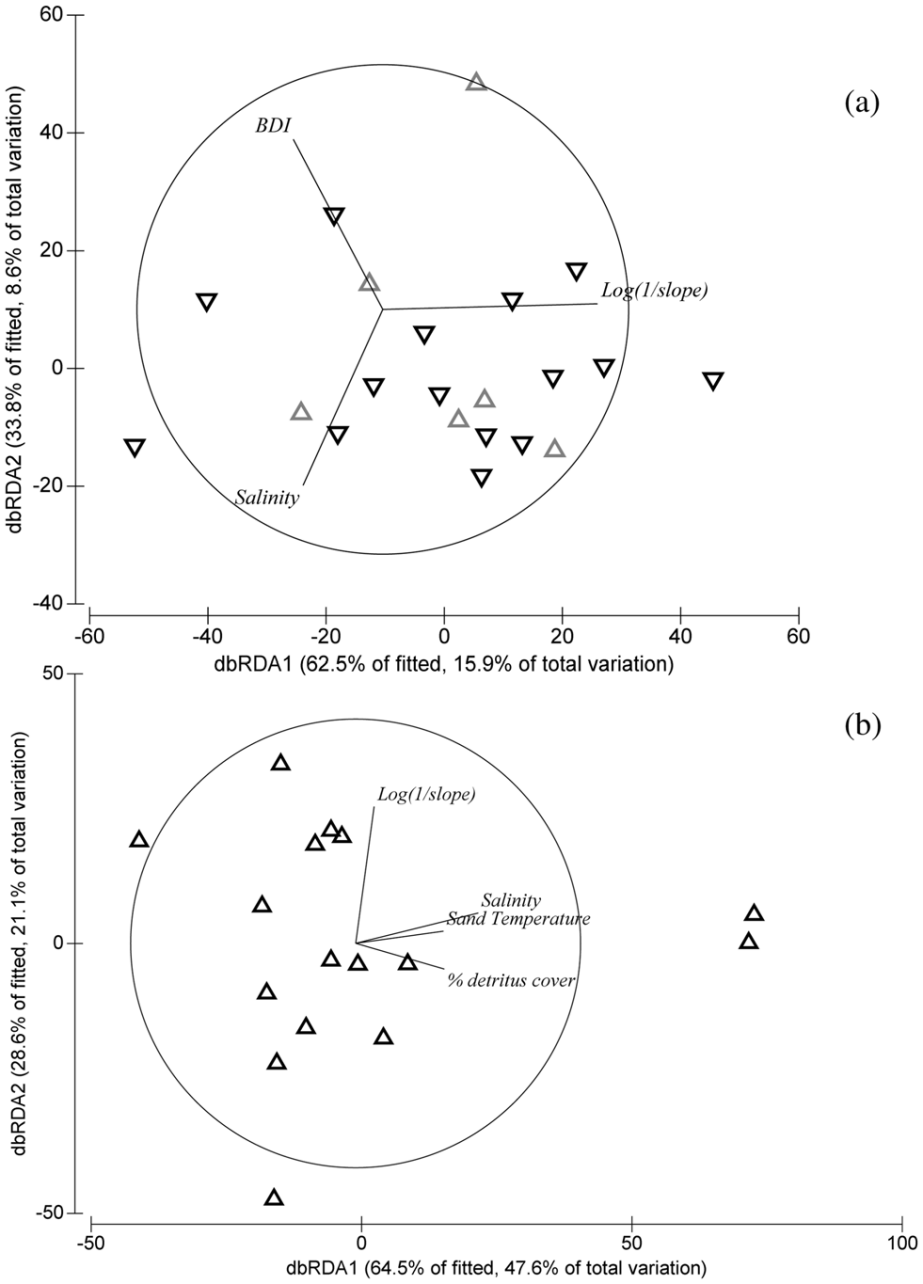
Distance Based Linear Model (DistLM) of abundance distribution along the southern African coasts of *Talorchestia capensis* (a) and *Africorchestia quadrispinosa* (b). The plots represent the absolute abundance data for each site of collection. The grey triangles show the sites in the Cool Temperate bioregion; the black triangles, the sites in the Warm Temperate bioregion. The dbRDA values are reported for the first (dbRDA1) and second axes (dbRDA2). The base variables which best explain the distribution of abundance are reported in the graph as vectors. (resemblance: Bray Curtis similarity; transformation: square root; correlation type: Pearson, correlation>0.2).

The results of the DistLM on the environmental factors showed that BDI, Slope, salinity, % of detritus and sand temperature were strong predictor variables in the distribution of the two species (Fig. 3). The DistLM showed the best results for *A. quadrispinosa* abundance distribution, with 68.7% of cumulative variation explained (dbRDA1 and dbRDA2). Higher priority should be given to the dbRDA1 axis than the dbRDA2 axis, with salinity having the stronger effect. Sand temperature and percentage of detritus cover hade a similar, but less strongly correlated relationship with abundance. The analyses also reported an effect of the log(1/slope) on the abundance data of *A. quadrispinosa*.

The DistLM showed that the best fit for *T. capensis* abundance and distribution was obtained using three predictor variables, though even combined, these explained very little of the variation in the data cloud (cumulative variation explained 24.5%) : BDI, log(1/slope) and salinity. Abundance was mostly explained by the dbRDA1 (21.1% of total variation) and among the three variables, log(1/slope) was the strongest predictor for *T. capensis.*

## Discussion

The Abundant Centre Hypothesis postulates the presence of an optimal centre of distribution, where species are more abundant, primarily because environmental requirements are assumed to be optimal in the centre, and degrade towards the margins [12], [17].

The ACH is based on the fact that both abundance and distribution are driven by biotic and abiotic environmental factors [17] and on the assumption that these environmental requirements are spatially auto correlated, so that sites close to one another are supposed to meet species requirements to a similar degree [10], [12]. Consequently, sites located far from the “optimal centre” are less likely to meet these requirements [10], [12], [17]. Sandy beaches are physically dominated systems and extremely variable in space and time [52]. The importance of the morphodynamic state of a beach on species richness, abundance, growth and reproduction is a debated argument [29]. The Swash Exclusion Hypothesis states that dissipative beaches have higher species richness, abundance and biomass than reflective ones, consequently a single site (i.e: a single beach) does not necessarily have the same characteristic as an adjacent one [64]. In contrast, the Habitat Safety Hypothesis, which separates supralittoral from intertidal forms, states that supralittoral species (such as sandhoppers) have higher abundances, individual growth, survival and reproduction rates on reflective than on dissipative beaches [29]. Furthermore Gomez & Defeo [30] found that supralittoral crustaceans increased in abundance from dissipative to reflective beaches in South America, a tendency opposite to that of intertidal animals, which increased from reflective to dissipative.

Considering these fundamental principles, we tested the ACH for the first time on sandhoppers. Our support for the ACH, tested using the distributions of abundance, size and sex ratio for sandhoppers, was equivocal and differed between the two species examined, with the strongest support coming from the most abundant and most widespread species. The analyses on the environmental variables confirmed the importance of the morphodynamic state of the beach as a fundamental driving-factor for the abundance of sandhoppers. Important here was the fact that, although all our shores were categorised as intermediate in state, within that category, beach slope still had a critical effect of the fauna.

### Africorchestia Quadrispinosa

Not surprisingly, *A. quadrispinosa* was the species that best fitted the model predictions. The large scale continuous distribution and the north-south orientation of its geographical range, provide a very suitable model to test the ACH.

The distribution of abundance and size of *A. quadrispinosa* followed a ramp-shaped pattern, with animals being more abundant towards the northern limits. Ramped patterns are generally attributed to unexpected changes in habitat or environmental conditions [12], [13], [65]. This could explain the rapid changes in abundance among several relatively closely positioned sites on the west coast of South Africa (from Cape Columbine, to Port Nolloth) and Namibia (from the border to Swakopmund, see Table S1 for GPS coordinates).

In contrast, the size distribution of *A. quadrispinosa* was south-ramped, with larger animals, both females and males, towards the southern edge of distribution. The relationship between size and range edges is highly debated, particularly in the case of endotherms, like mammals and birds (e.g. [15]). Some studies suggest that larger individuals occur at the core of the range (in agreement with the ACH) where the habitat is most suitable [11], [12], [66]. Alternatively, individuals tend to be larger towards higher latitudes. Populations distributed along a North-South axis therefore tend to show larger individuals near one of the edges rather than the core [66], [67]. Our results, confirmed this last tendency of larger size at higher latitudes, with *A. quadrispinosa* showing a ramped south distribution of size.

### Talorchestia Capensis

The size distribution of female *T. capensis*, was best fitted by an Inverse Quadratic model, providing positive support for a centre pattern hypothesis. Females of *T. capensis* were larger in size on temperate sandy beaches than in the sub-tropical and cool-temperate biogeographic regions, a trend also found for sandy shore isopods by Cardoso & Defeo [68]. In general, size is positively related to food availability and quality, which should support the ACH [15]. The interaction between beach morphodynamics and sandhopper size and density is usually positively correlated, with dissipative and temperate beaches offering a more suitable habitat, even though a reverse trend has been showed (an increase of size and a decrease of density towards reflective beaches type) for supralittoral crustaceans, in several sandy beaches in South America [29], [69]. The results of the DistLM for *T. capensis*, suggest an influence of the Slope, BDI and salinity on abundance. Beach morphodynamics interact very tightly with the amount of detritus, with beach morphodynamic state being the fundamental driver operating through its effect on food availability. Two substantial gaps appeared in the distribution of *T. capensis*, making it discontinuous. A gap on the south-coast is explained by the absence of suitable habitat as this stretch of coast forms continuous rocky shores. The 600 km gap on the west coast is more difficult to explain as it includes stretches of sandy shore. Sampling over such a large geographic scale necessarily provides only a snapshot of abundances (although the same pattern of distribution as the present one was confirmed by collections for genetic analysis along the entire coastline during May 2012) and temporal variation could explain unexpected absences from sites. It is not uncommon for sandhoppers to show seasonal changes in their within-shore distribution, as well as geographic differences. Tsubokura et al. [70], found that sandhoppers burrow more deeply and farther inland during winter, migrating down shore and burrowing less deeply during spring [70]. In general talitrids are concentrated along the high tide mark, burrowed underneath the largely macrophytes detritus on the shore [70], although on the south coast of South Africa *T. capensis* can show higher abundances towards the dune base or even into the dune slacks rather than in the intertidal [71].

Information on population genetics could help clarify whether the observed gaps were artifactual by explaining the degree of isolation between population centres. Preliminary results indicate high genetic diversity among several populations (SB, unpubl. data).

### Conclusions

In general, the predictions of the ACH gained little support from the observed data and, consequently, the hypothesis of a general model of an optimal centre of distribution of abundance, size, and sex ratio must be broadly rejected for these southern African sandhoppers. Indeed conformation with the predictions of the ACH has been described as “more the exception than the rule”, [10, *p.993*] and is often considered to over-simplify species distributions [65] or to work only for a north-south range of distribution [14], [16]. Nevertheless, our most suitable test organism, *A. quadrispinosa*, provided the strongest support for the predictions of the ACH. This suggests that the ACH may be applicable, but only in certain cases where organisms are abundant and show clear patterns of distribution. An accepted tool for conservation planning is the development of distribution models able to evaluate species ranges in relation to environmental changes [2], [72]. The present study reported a mesoscale investigation of the biogeography of supralittoral amphipods, which contribute to the biomass of wrack associated macrofauna of sandy beaches and therefore play an important role in the bottom up trophic ecology of sandy beaches [73]. Understanding what regulates the boundaries of species range is crucial, especially given predictions of accelerated environmental change [11] and is particularly relevant for these systems as they are highly dynamic and respond strongly to environmental forcing [73]. Environmental effects operate hierarchically and changes perceived by individuals need not be reflected in the dynamics of a species’ biogeography. Individual plasticity, might therefore be a key factor when investigating the links between the environment and the distribution of organisms [11], [13], [14], [16], [22].

The DistLM analyses, showed that, even within the category of intermediate morphodynamic state, beach slope was particularly important. This is central as it offers an explanation for the weak support gained by to the ACH: individual stretches of beach are highly differentiated from one another, mainly due to physical differences. This is in contrast with the main assumption of the ACH that sites close to one another should provide similar environmental conditions [12].

The DistLM also showed an important effect of salinity on the distribution of the abundance for both species. Salinity is a fundamental factor for sandhoppers as they have colonised terrestrial environments, which requires extreme physiological adaptations [74]. A concurrent variations in salinity and sedimentological variables are fundamental in shaping the spatial distribution of abundance in sandy beach macrofauna. [75].

Although *T. capensis* abundance occurs on both the west and east coasts, encompassing two biogeographic regions with widely different temperature regimes, the distribution of *A. quadrispinosa* suggests that temperature is an important factor in shaping distributions and range limits as its southern limit of distribution ends in an area which is often considered as a transition between the cool temperate and warm temperate regions [33], [35]. In accord with this, sand temperature was linked to the distribution of *A. quadrispinosa* abundance in the DistLM. Nevertheless, temperature generally had little effect in these analyses and this could be attributed to our methodology which provided only an instantaneous measure of temperature which is a much less integrated variable than salinity. Further investigations of the effects of temperature should include an integrate estimate of temperature using temperature data loggers, if possible [76].

Further analysis of the thermal tolerances of these species will help clarify the complex dynamics that drive their biogeographic ranges and make predictions of how these may shift.

Despite the weak support for the ACH provided by these sandhoppers, the importance of environmental parameters in driving their distributions was clear, especially in systems and spatial scales where the abundance of animals is more related to physical than biological factors. One limiting and simplistic aspect of this and other tests of the ACH is the focus on adult organisms and it is possible that the integration of environmental effects on different life stages and reproduction (which might generate maternal effects), would clarify the synergies and/or constraints that result in the distribution of organisms along latitudinal gradients.

## Supporting Information

Figure S1 **Scheme showing the sampling design used at each sampling site.** The four traps has been set at the corner of the two baffles in order to maximize the collection and retrieve information on the orientation of the migratory activities (unpublished data). The traps has been named has follows: n = north; s = south; e = east; w = west. The position and the name of the traps do not coincide with the cardinal points, but the arrangement is purely related to the position relative to the shore. For instance, the trap named “n” is the one facing the dunes, while the “s” is toward the swash line. Consequently, the traps “e” and “w” are set, respectively at the right and left of the X arrangement. The two separated levels were assessed in order to investigate pattern of abundance at microscale and the effect of new fresh detritus (normally occurring at the L2) on the zonation of juveniles and ovigerous females (unpublished data).
(TIF)

Figure S2 **Five Hypothetical models proposed for explain the patterns of distribution of abundance, size and sex ratio along the geographical range of the two species of sandhoppers (modified from** **[10]**, **[16**] **).** Normal model (a), Inverse Quadratic (b), Ramped South (c), Ramped North (d), Abundant edges (e). We calculated the residual sum of square deviations (RSS, deviations indicated by arrows) for the observed data that exceeded the constraint boundary (open dots). The grey dots represent the analysed trait values.
(TIF)

Table S1 **List of the sampling sites with GPS coordinates and bioregions of interest (following** **[33]** **).**
(RTF)
