# Supplementary figures and images for: Environmental Domains and Range-Limiting Mechanisms: Testing the Abundant Centre Hypothesis Using Southern African Sandhoppers
Source: PLoS One. 2013 Jan 23;8(1):e54598. doi: 10.1371/journal.pone.0054598 (PMC3553053; doi:10.1371/journal.pone.0054598)

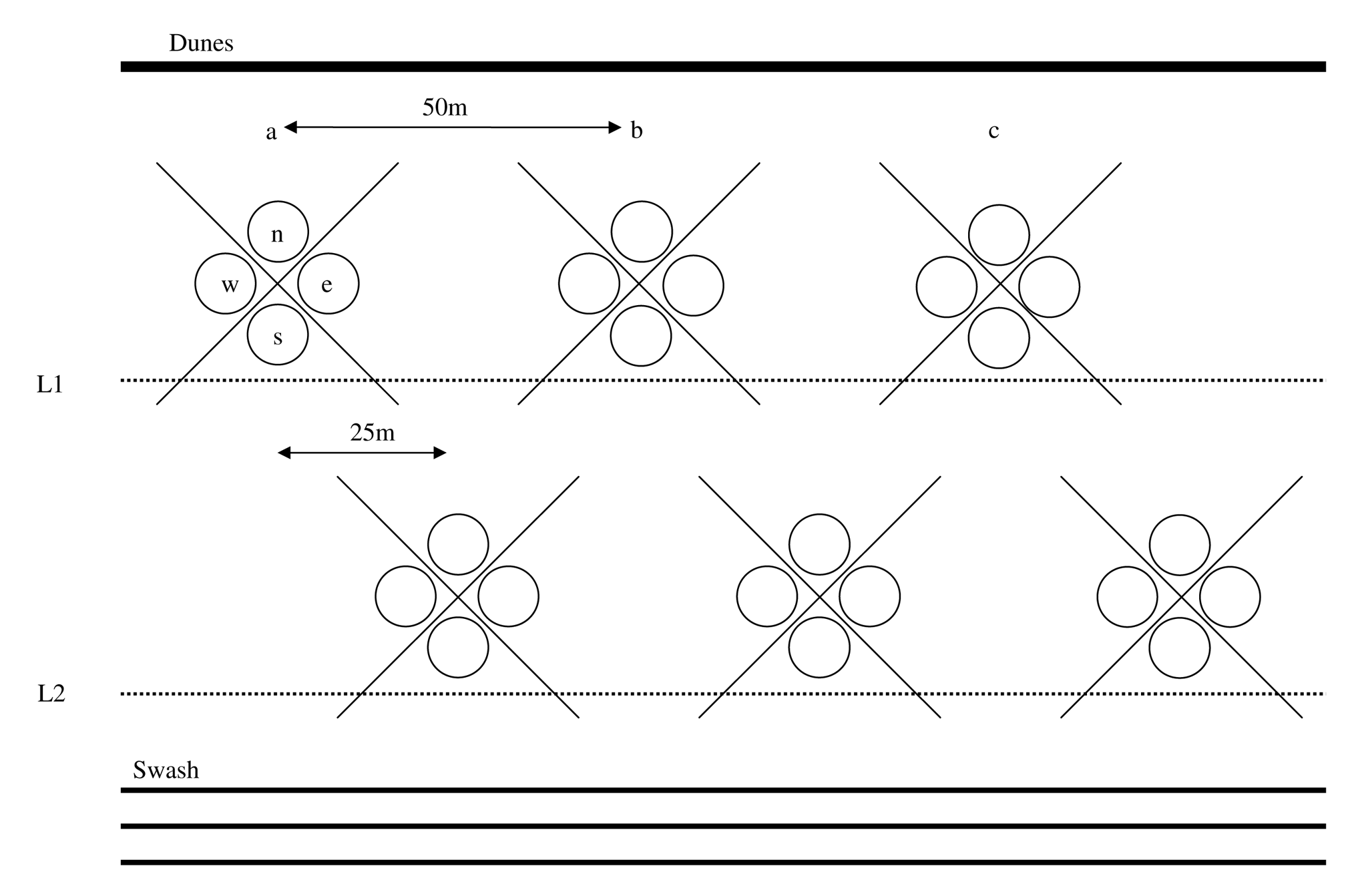

Supplement: Figure S1 — Scheme showing the sampling design used at each sampling site. The four traps has been set at the corner of the two baffles in order to maximize the collection and retrieve information on the orientation of the migratory activities (unpublished data). The traps has been named has follows: n = north; s = south; e = east; w = west. The position and the name of the traps do not coincide with the cardinal points, but the arrangement is purely related to the position relative to the shore. For instance, the trap named “n” is the one facing the dunes, while the “s” is toward the swash line. Consequently, the traps “e” and “w” are set, respectively at the right and left of the X arrangement. The two separated levels were assessed in order to investigate pattern of abundance at microscale and the effect of new fresh detritus (normally occurring at the L2) on the zonation of juveniles and ovigerous females (unpublished data). (TIF) [file pone.0054598.s001.tif]

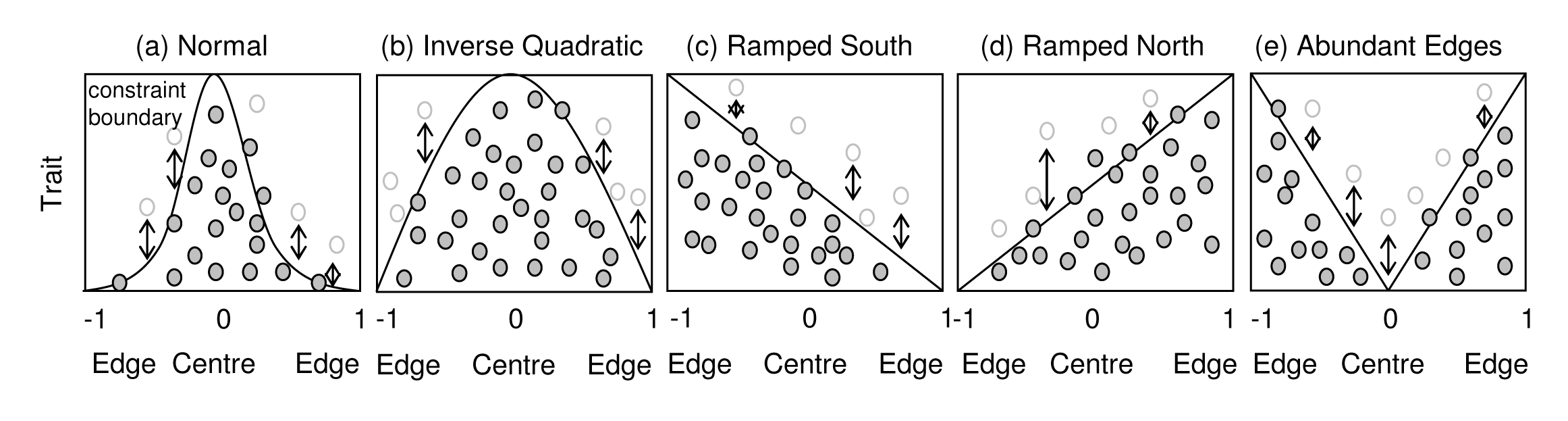

Supplement: Figure S2 — Five Hypothetical models proposed for explain the patterns of distribution of abundance, size and sex ratio along the geographical range of the two species of sandhoppers (modified from [10] , [16] ). Normal model (a), Inverse Quadratic (b), Ramped South (c), Ramped North (d), Abundant edges (e). We calculated the residual sum of square deviations (RSS, deviations indicated by arrows) for the observed data that exceeded the constraint boundary (open dots). The grey dots represent the analysed trait values. (TIF) [file pone.0054598.s002.tif]
