# Supplementary material for: Environmental Domains and Range-Limiting Mechanisms: Testing the Abundant Centre Hypothesis Using Southern African Sandhoppers
Source: PLoS One. 2013 Jan 23;8(1):e54598. doi: 10.1371/journal.pone.0054598 (PMC3553053; doi:10.1371/journal.pone.0054598)
Supplement: Table S1 — List of the sampling sites with GPS coordinates and bioregions of interest (following [33] ). (RTF) [file pone.0054598.s003.rtf]

Table S1. List of the sampling site with GPS coordinates and bioregions of interest (following [33])
Site	Coordinate	Bio-region	
Swakopmund	S22.67410 E14.52784	Cool-temperate	
Luderitz	S26.64583  E15.153889	Cool-temperate	
Port Nolloth	S29.28002 E16.87979	Cool-temperate	
Hodenklipbaai	S30.31644 E17.27566	Cool-temperate	
Groenrivier	S30.59690 E17.44224	Cool-temperate	
Doringbaai	S31.74402 E18.22239	Cool-temperate	
C. Columbine	S32.87481 E17.88049	Cool-temperate	
Yzerfontein	S33.34021 E18.16109	Cool-temperate	
Blouberstand	S33.85028 E18.48831	Cool-temperate	
Muizenberg	S34.09897 E18.49471	Warm-temperate	
Pringle Bay	S34.33435 E18.82616	Warm-temperate	
Kleinmonde	S34.34219 E19.04712	Warm-temperate	
Mosselrivier	S34.41525 E19.28987	Warm-temperate	
Franskraal	S34.61053 E19.42179	Warm-temperate	
Pearly Beach	S34.66928 E19.51424	Warm-temperate	
Struiss Bay	S34.78577 E20.04620	Warm-temperate	
Still Bay	S34.37306 E21.42896	Warm-temperate	
Mossel Bay	S34.05003 E22.30704	Warm-temperate	
Knysna	S34.08062 E22.97628	Warm-temperate	
Jeffreys Bay	S33.96930 E25.01428	Warm-temperate	
Sundays River	S33.78453 E25.36834	Warm-temperate	
Port Alfred	S33.89336 E26.29815	Warm-temperate	
Kidd's Beach	S33.92725 E27.87636	Warm-temperate	
Kei Mouth	S32.68138 E28.38368	Warm-temperate	
Port St. Johns	S31.62035 E29.55922	Subtropical	
Port Edward	S31.03222 E30.23677	Subtropical	
Clansthal	S30.24457 E30.78153	Subtropical	
Ballito	S29.61103 E33.59488	Subtropical	
Richard's Bay	S29.09553 E32.44105	Subtropical	
